# Supplementary material for: Comparative Evaluation of Analgesics in a Murine Bile Duct Ligation Model
Source: Biomedicines. 2025 Dec 10;13(12):3034. doi: 10.3390/biomedicines13123034 (PMC12730638; doi:10.3390/biomedicines13123034)
Supplement: Supplementary file 1 [file biomedicines-13-03034-s001.zip › biomedicines-4001844-supplementary.pdf]

Supplementary Table S1. Distress score sheet

Observation	Score
**I Body weight**	
I-a decreased > 10% (compared to initial weight)	2
I-b decreased > 20% (compared to initial weight)	5
**II General condition**	
II-a tooth displacement, too long teeth	1 (A)
II-b fur dull, ruffled or untended	2
II-c eyes unclear or squinted	2
II-d untended orifices of the body	3
II-e abnormal posture	3
II-f dehydration	3
II-g short spasms or temporary paralysis symptoms	3
II-h persistent (>30’) cramping or paralysis	5
II-i abnormal respiratory sounds or animal feels cold	5
**III Spontaneous behavior**	
III-a the animal is passive or overactive	2
III-b pronounced apathy, hyperkinetic, or isolation	4
III-c squeaking due to pain	5
III-d self-mutilation	5
**IV Flight behavior after contact**	
IV-a animal is passive or overactive	2
IV-b distinct apathy or hyperkinetic	5
**V Process-specific criteria**	
V-a wound healing disorder	2
V-b opening of the sutures by biting	1 (B)
V-c local inflammation	2
V-d ascites	4
Total score	0-66

Score points are stated per line as soon as one criteria applies. Even with several positive results per line, there is no addition of the points per line.

Supplementary Table S2. Consequences according to distress score.

Single score	Total score	Distress level	Measures
A		mild	Anesthetize animal and shorten teeth. Document it.
B		mild	Inform the person in charge of the experiment. If necessary, anesthetize the animal and close the wound. Document it.
1		mild	Inform the person in charge of the experiment. A sufficient frequency of observation is necessary, consider treatment options and document it.
2-4		moderate	Inform the person in charge of the experiment. Daily observation of the animal is necessary, consider treatment options and document it.
5		severe*	In agreement with the person in charge euthanasia (preferably painless after anesthesia) has to be performed. Document it.
	3-4	mild	Inform the person in charge of the experiment. Daily observation of the animal is necessary, consider treatment options and document it.
	5-15	moderate	Inform the person in charge of the experiment. Euthanasia or treating the animal plus daily observation of the animal is necessary. Document it.
	>15	severe*	In agreement with the person in charge euthanasia (preferably painless after anesthesia) has to be performed. Document it.

Note:

- without euthanasia the animals would experience severe distress
- body weight decreased > 25% (compared to initial weight) the consequence of euthanasia takes place

Supplementary Table S3. Calculated doses of ingested metamizole (mg/kg/24 h) for each mouse on each day.

Day	calculated dose of ingested metamizole (mg/kg/24h)									n below 150 mg/kg [1] (lowest threshold)
	Mouse 4	Mouse 6	Mouse 9	Mouse 12	Mouse 15	Mouse 20	Mouse 21	Mouse 22	Mouse 26	
**Pre**	442.31	402.99	415.38	527.47	406.78	307.69	335.53	369.57	380.28	0
**A1**	327.97	228.48	305.88	253.52	348.12	180.45	165.52	229.79	285.71	0
**A2**	281.88	280.00	318.18	307.17	359.15	180.45	222.22	223.88	236.56	0
**-22**	98.36	87.59	228.14	62.72	40.68	67.92	19.80	67.42	221.40	7
**-21**	288.89	47.43	413.79	165.35	293.23	301.26	0.00	376.57	237.15	2
**-15**	352.94	412.37	505.75	191.49	437.96	390.24	40.13	342.11	407.55	1
**-8**	378.95	272.73	400.00	235.71	291.67	336.00	81.36	463.32	397.06	1
**-2**	456.75	294.74	445.31	296.82	312.5	338.71	202.02	320.61	320.28	0
**0**	81.08	62.50	46.51	21.51	61.02	305.88	21.98	45.45	107.14	8
**1**	203.77	0.00	231.76	142.86	23.17	215.25	22.90	49.18	148.15	6
**4**	457.83	106.67	398.23	307.69	618.83	625.59	73.77	/	497.82	2
**7**	/	204.26	672.90	314.52	515.02	270.27	/	/	467.53	0
**12**	/	209.61	113.21	419.75	485.11	230.77	/	/	774.19	1

Supplementary Table S4. Calculated doses of ingested tramadol (mg/kg/24 h) for each mouse on each day.

Day	calculated dose of ingested tramadol (mg/kg/24h)										n below 20 mg/kg [2] (lowest threshold)
	Mouse 2	Mouse 3	Mouse 5	Mouse 10	Mouse 11	Mouse 17	Mouse 18	Mouse 23	Mouse 27	Mouse 30	
**Pre**	136.69	126.76	161.40	144.83	150.54	139.19	144.49	129.25	130.91	108.47	0
**A1**	113.88	201.39	98.94	147.37	120.57	194.03	134.83	68.03	107.91	82.76	0
**A2**	116.79	143.37	117.22	134.75	113.07	115.12	111.52	116.44	139.71	97.56	0
**-22**	79.14	106.38	145.45	99.29	56.54	43.64	75.76	123.71	29.41	28.17	0
**-21**	146.72	206.90	137.93	154.41	146.72	88.89	124.48	160.31	98.77	122.14	0
**-15**	135.85	138.18	177.61	178.57	124.54	134.83	181.10	164.29	222.22	159.09	0
**-8**	152.09	194.95	184.62	152.25	152.73	164.75	166.00	178.57	193.05	148.15	0
**-2**	137.93	173.91	160.31	154.93	136.69	151.52	140.08	170.82	151.52	125.00	0
**0**	126.39	122.74	30.08	21.13	14.08	106.06	46.15	22.56	44.78	7.38	2
**1**	213.68	131.87	91.60	95.24	148.15	119.52	192.14	41.67	99.59	72.29	0
**4**	150.44	157.30	/	/	245.45	136.17	159.62	133.33	142.26	198.20	0
**7**	229.36	178.44	/	/	198.20	125.00	240.38	/	157.68	183.41	0
**12**	188.68	146.52	/	/	102.80	110.60	158.42	/	123.22	104.00	0

Supplementary Table S5. Calculated doses of ingested carprofen (mg/kg/24 h) for each mouse on each day.

Day	calculated dose of ingested carprofen (mg/kg/24h)									n below 20 mg/kg [3] (lowest threshold)
	Mouse 1	Mouse 7	Mouse 8	Mouse 13	Mouse 16	Mouse 19	Mouse 24	Mouse 25	Mouse 28	
**Pre**	23.96	20.06	20.07	24.72	17.76	16.73	21.58	29.00	16.89	3
**A1**	25.95	18.45	21.43	23.42	19.8	17.27	25.56	33.21	19.93	4
**A2**	20.03	15.05	19.85	14.29	18.94	17.58	17.98	26.54	17.94	7
**-22**	13.31	21.77	26.34	14.79	16.11	10.87	13.48	14.61	14.33	7
**-21**	21.19	14.85	18.18	28.13	25.88	16.41	19.39	25.51	14.74	5
**-15**	24.64	23.39	24.14	33.33	21.98	18.39	24.72	28.74	23.32	1
**-8**	24.73	17.11	26.64	17.39	18.28	15.85	24.54	24.23	23.00	4
**-2**	25.00	23.47	22.64	19.22	19.78	15.85	25.65	28.52	24.91	3
**0**	6.29	5.07	8.05	7.45	9.85	9.02	26.47	2.29	15.73	8
**1**	4.65	10.11	15.13	14.46	12.50	8.50	21.76	0	12.77	8
**4**	/	20.17	/	18.58	/	20.64	29.74	/	20.3	1
**7**	/	18.91	/	33.19	/	/	26.51	/	17.79	2
**12**	/	209.61	/	29.73	/	/	27.01	/	21.89	0

Supplementary Table S6. Number of animals contributing data at each experimental day.

days	animals present in the study on each experimental day		
	metamizole	tramadol	carprofen
**Pre**	10	10	9
**A1**	10	10	9
**A2**	10	10	9
**-22**	10	10	9
**-21**	10	10	9
**-15**	10	10	9
**-8**	10	10	9
**-2**	10	10	9
**0**	10	10	9
**1**	10	10	9
**4**	9	8	6
**7**	7	8	4
**12**	7	7	3

References

1. Stumpf, F.; Algül, H.; Thoeringer, C.K.; Schmid, R.M.; Wolf, E.; Schneider, M.R.; Dahlhoff, M. Metamizol Relieves Pain Without Interfering With Cerulein-Induced Acute Pancreatitis in Mice. *Pancreas* **2016**, *45*, 572–578, doi:10.1097/MPA.0000000000000483.
2. Sacerdote, P.; Bianchi, M.; Manfredi, B.; Panerai, A.E. Effects of tramadol on immune responses and nociceptive thresholds in mice. *Pain* **1997**, *72*, 325–330, doi:10.1016/s0304-3959(97)00055-9.
3. Matsumiya, L.C.; Sorge, R.E.; Sotocinal, S.G.; Tabaka, J.M.; Wieskopf, J.S.; Zaloum, A.; King, O.D.; Mogil, J.S. Using the Mouse Grimace Scale to reevaluate the efficacy of postoperative analgesics in laboratory mice. *J. Am. Assoc. Lab. Anim. Sci.* **2012**, *51*, 42–49.
